# Synthetic phase sensitive inversion recovery late gadolinium enhancement from post-contrast T1-mapping shows excellent agreement with conventional PSIR-LGE for diagnosing myocardial scar

**DOI:** 10.1186/1532-429X-16-S1-P213

**Published:** 2014-01-16

**Authors:** Goran Abdula, Peder Sörensson, Magnus Lundin, Jannike Svedin, Margareta Klein, Peter Kellman, Andreas Sigfridsson, Martin Ugander

**Affiliations:** 1 Karolinska Institutet, Stockholm, Sweden; 2 NIH, Bethesda, Maryland, USA

## Background

Cardiac magnetic resonance (CMR) imaging using phase sensitive inversion recovery (PSIR) late gadolinium enhancement (LGE) is the in vivo reference standard for assessing focal myocardial scar. Post-contrast T1-mapping by Modified Look-Locker Inversion recovery (MOLLI) can be used to generate a Synthetic PSIR LGE (SynLGE) image with an image contrast similar to conventional LGE images. We aimed to identify focal myocardial scar by SynLGE and compare its diagnostic accuracy with the reference standard LGE. We hypothesized that SynLGE has an accuracy which approaches LGE for diagnosing focal myocardial scar.

## Methods

Consecutive patients (n = 60, mean ± SD age 50 ± 17 years, 60% male) referred for clinical CMR at 1.5T (Siemens Avanto or Aera) received an intravenous contrast bolus (0.2 mmol/kg gadoteric acid, Gd-DOTA, Dotarem^®^). PSIR LGE (1.3 × 1.3 × 8 mm) and post-contrast MOLLI (1.4 × 1.4 × 6 mm, 5-3 acquisition scheme, motion corrected) were acquired starting 10-15 and 20-25 minutes post bolus, respectively. A cardiac short-axis stack and three long-axis views were acquired for SynLGE and LGE. SynLGE at TI 300 ms were generated off-line from post-contrast T1-maps, and an in-line version is now available. Only LGE and SynLGE images (no cine images) were analyzed by two blinded observers for agreement regarding localization and origin of myocardial scar on a per-patient basis. Identification of scar required confirmation in two contiguous or orthogonal slices.

## Results

**Figure 1:**
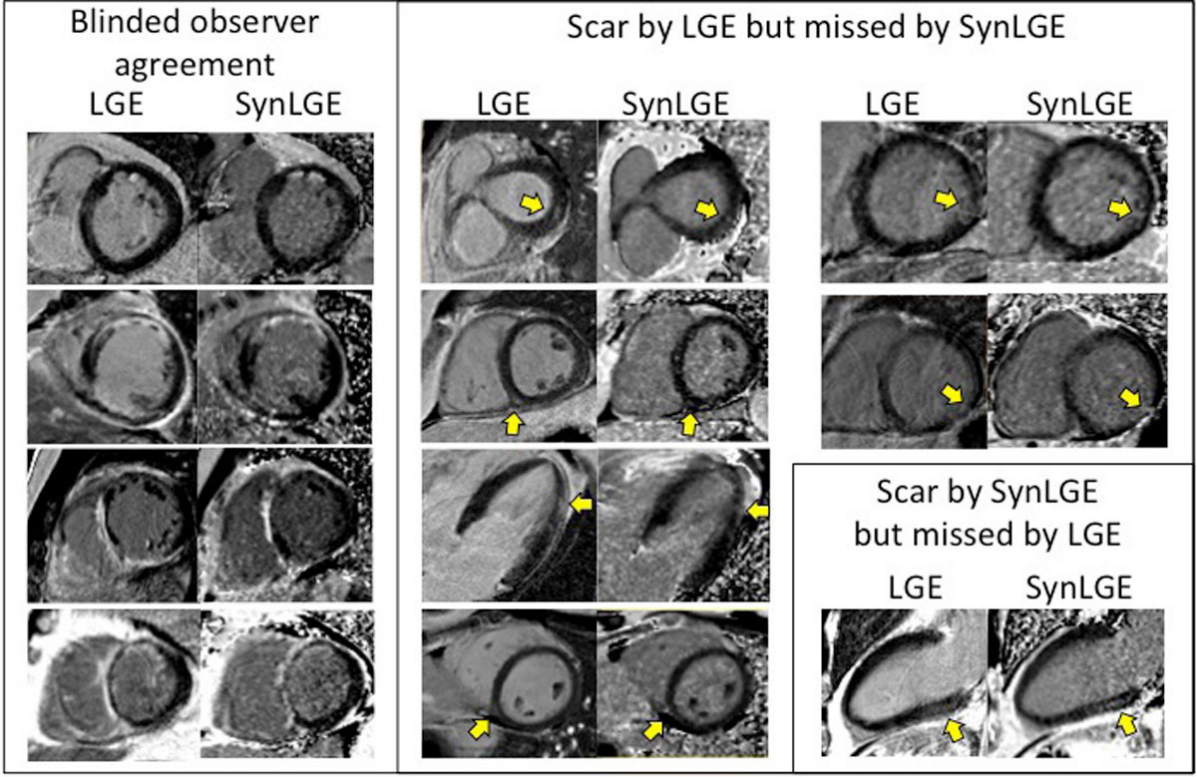


Consensus identified scar by LGE in 17/60 (28%) patients. The interobserver disagreement prior to consensus was 11/60 (12%). Scar patterns were non-ischemic (n = 9), ischemic (n = 6) or both (n = 2). Compared to LGE, SynLGE yielded a diagnostic sensitivity of 17/23 (74%), specificity of 36/37 (97%), positive predictive value of 17/18 (94%), negative predictive value of 36/42 (86%), and an overall accuracy of 53/60 (88%). In cases where SynLGE missed a scar (n = 6), these were either small non-ischemic scars (n = 4) or infarction in a thin myocardial wall (n = 2). In one case, SynLGE identified a midmural non-ischemic scar not identified by LGE. See Figure 1.

## Conclusions

Overall, SynLGE showed high agreement with LGE. There were fewer differences between consensus LGE and SynLGE than there were differences in interobserver comparison for LGE, suggesting clinical equivalence of LGE and SynLGE. Thus, conventional LGE may not be necessary when full left ventricular coverage post-contrast T1-maps with SynLGE are routinely acquired for myocardial extracellular volume assessment. SynLGE therefore has the potential to reduce total scan time, except in challenging cases where complementary LGE can be added.

## Funding

Swedish Research Council, Swedish Heart and Lung Foundation, Stockholm County Council, Karolinska Institutet, Swedish Medical Assocation, Swedish Society of Medical Research, and The Kleberg, Osterman, Wiberg, Erling-Persson, and Grönberg Foundations.

